# Supplementary material for: RIPK1 dephosphorylation and kinase activation by PPP1R3G/PP1γ promote apoptosis and necroptosis
Source: Nat Commun. 2021 Dec 3;12:7067. doi: 10.1038/s41467-021-27367-5 (PMC8642546; doi:10.1038/s41467-021-27367-5)

RIPK1 dephosphorylation and kinase activation by PPP1R3G/PP1γ promotes apoptosis and necroptosis

Jingchun Du[1,2,9], Yougui Xiang[1,3,9], Hua Liu[1,4], Shuzhen Liu[1], Ashwani Kumar[5], Chao Xing[5,6,7], Zhigao Wang[1,8*]

[1]Department of Molecular Biology, University of Texas Southwestern Medical Center, 5323 Harry Hines Boulevard, Dallas, TX 75390, USA

[2]Department of Clinical Immunology, Kingmed School of Laboratory Medicine, Guangzhou Medical University, Guangzhou, Guangdong 510182, China

[3]Caris Life Sciences, 4610 South 44th Place, Phoenix, AZ 85040, USA

[4]School of Pharmacy, Jiangxi University of Chinese Medicine, Nanchang, Jiangxi 330006, China

[5]Eugene McDermott Center for Human Growth and Development, University of Texas Southwestern Medical Center, 5323 Harry Hines Boulevard, Dallas, TX 75390, USA

[6]Department of Bioinformatics, University of Texas Southwestern Medical Center, 5323 Harry Hines Boulevard, Dallas, TX 75390, USA

[7]Department of Population and Data Sciences, University of Texas Southwestern Medical Center, 5323 Harry Hines Boulevard, Dallas, TX 75390, USA[8]Current address: Heart Institute, Department of Internal Medicine, University of South Florida, 560 Channelside Drive, Tampa, FL 33602, USA

[9]These authors contribute equally

*Correspondence: zhigao@usf.edu

Supplementary figure 1

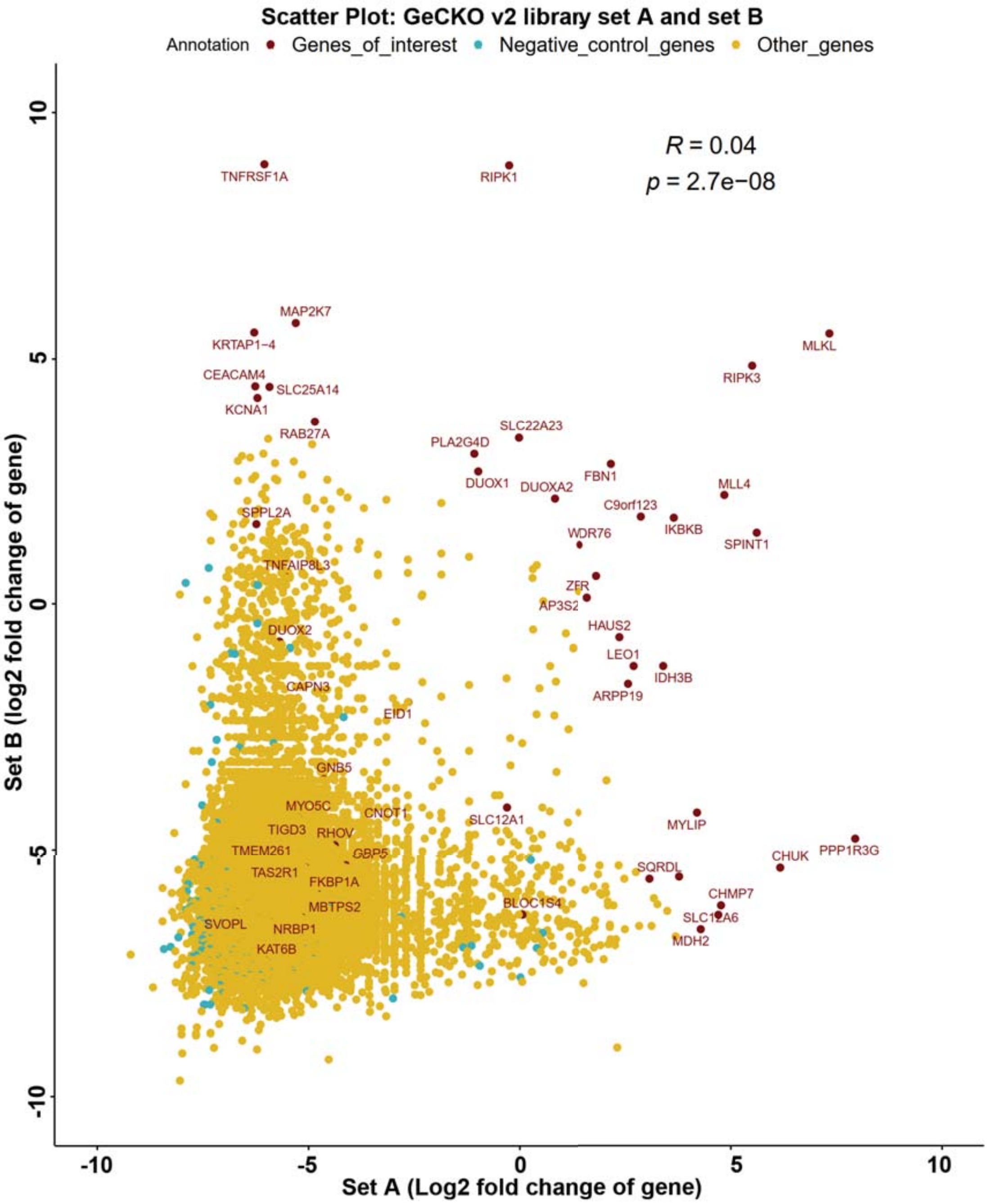

Supplementary Figure 1. Scatter plot for GeCKO v2 library set A and B. The GeCKO v2 library contains two sets of sgRNA for each gene, including 3 sgRNAs in library set A and 3 sgRNAs in library set B. Fold change between control and T/S/Z sample was calculated for each gene in set A and set B using MAGeCK and then plotted as a scatter plot. Spearman correlation coefficient and significance of correlation is shown in the plot. We first randomly subsetted the negative control guides into dummy genes. We then used MAGeCK test command with input options “control-sgrna option” and “norm-method control” (https://sourceforge.net/p/mageck/wiki/QA/#what-does-the-control-sgrna-control_sgrna-option-do-how-to-use-this-option). MAGeCK uses the list of negative control guides to generate the null distribution of RRA score during p-value calculation. As per the recommendation from authors of MAGeCK we used 1000 negative control guides for p value estimation. P-value significance of 2.7e-08 was found between set A and set B.

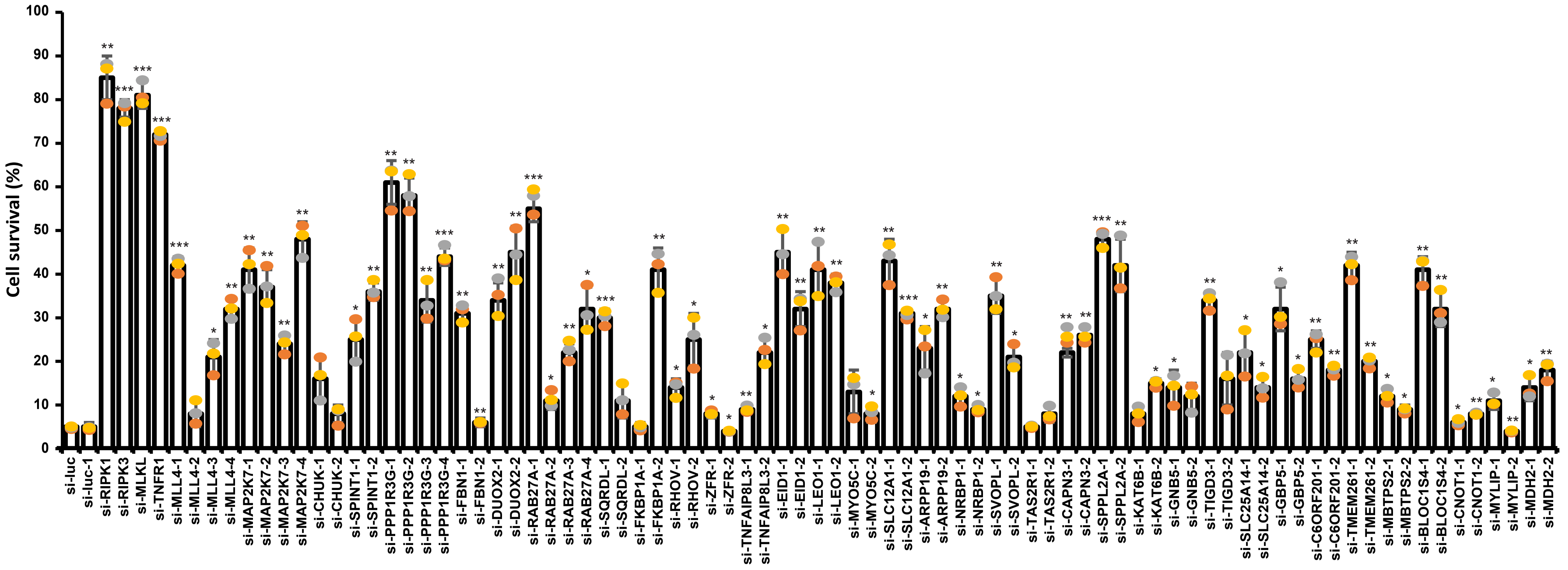

Supplementary Figure 2. Secondary siRNA screen. Small interfering RNAs (siRNA) were transfected into HAP1:RIPK3:MLKL cells for 48 hours followed by DMSO or T/S/Z treatment for 16 hours. Cell survival was measured with CellTiter-Glo assay. Viable cells expressed as a percentage of DMSO-treated cells. Results are represented as mean ± SD of n=3 biological independent samples. Two-tailed Student's t-test, *P<0.05; **P<0.01; ***P<0.001.

Supplementary figure 3. Source data immunoblots.

Fig. 1a

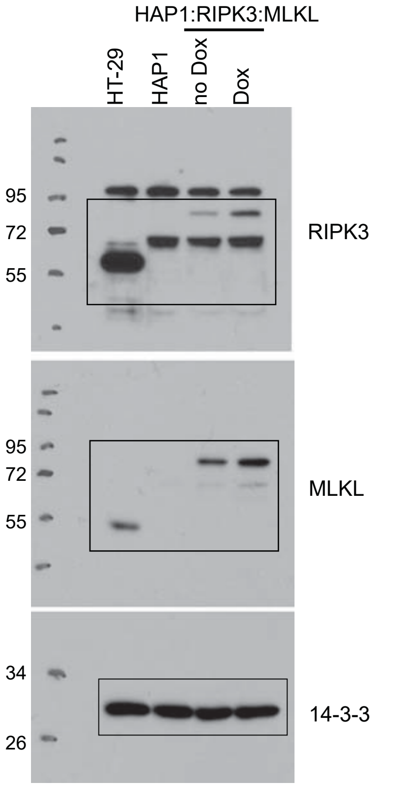

Fig. 2j

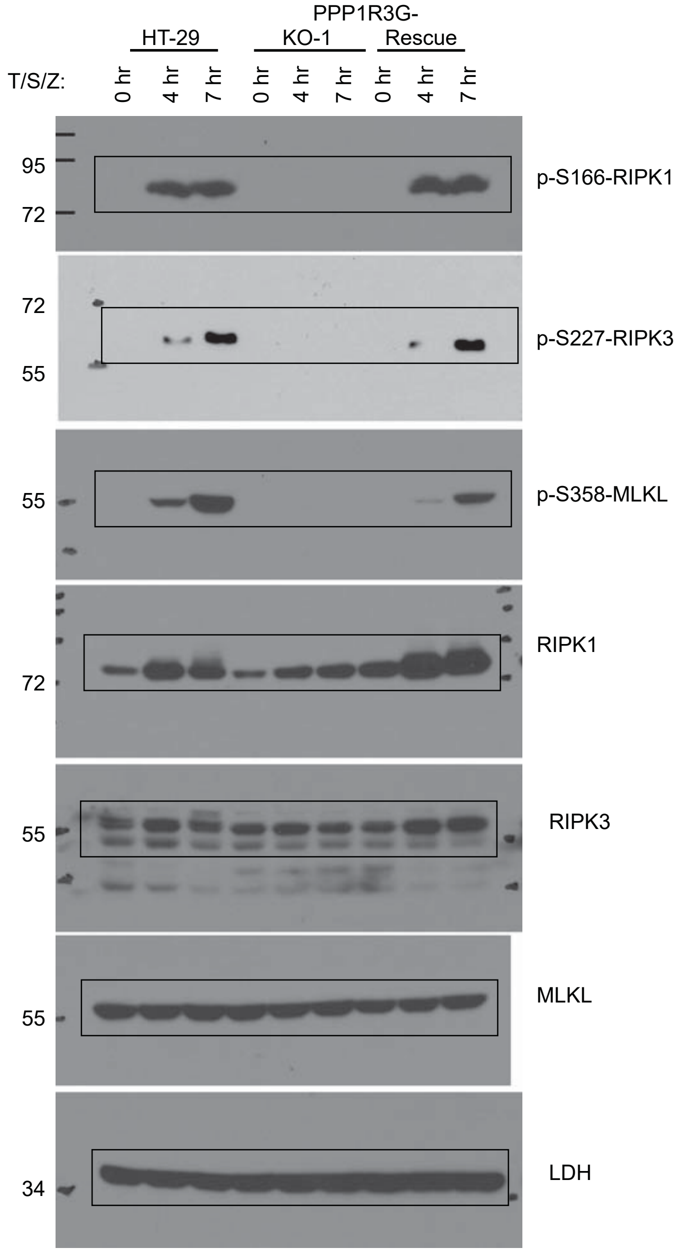

Fig. 2c

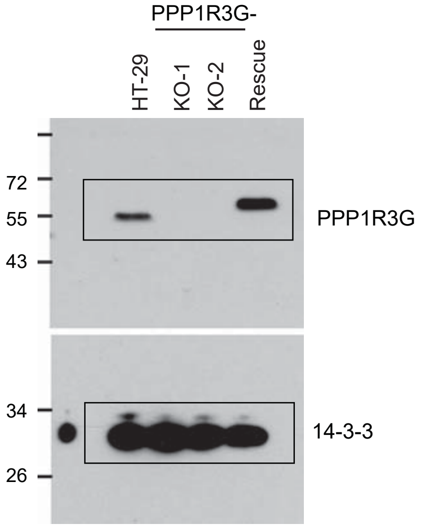

Supplementary figure 3. Source data immunoblots (continued).

Fig. 3b

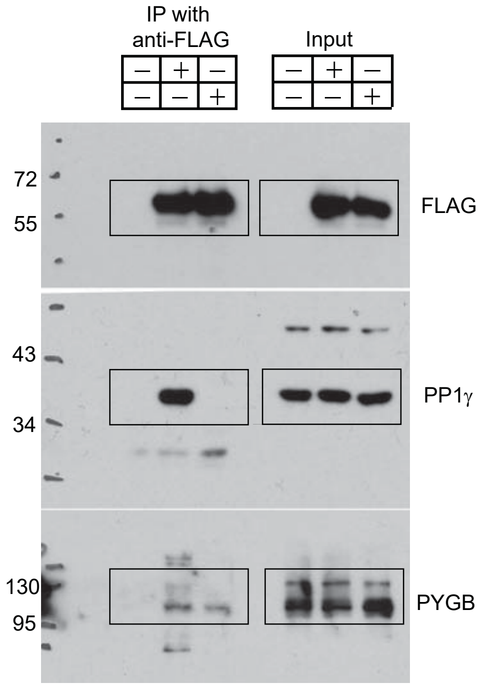

Fig. 3d

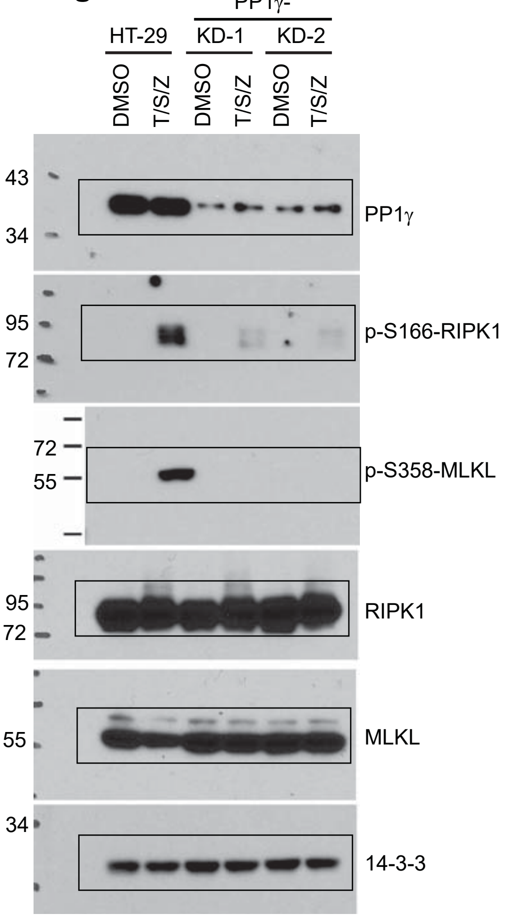

Fig. 3e

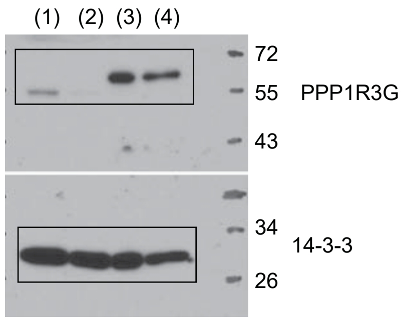

Fig. 3f

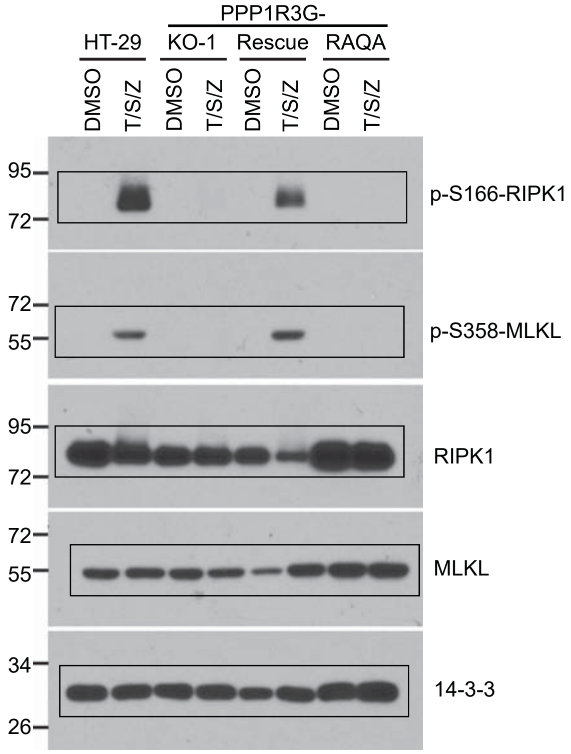

Fig. 3h

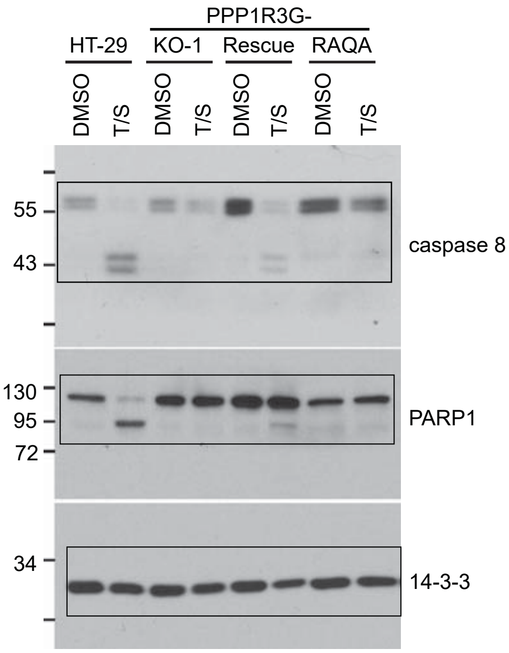

Supplementary figure 3. Source data immunoblots

(continued). **Fig 4a**

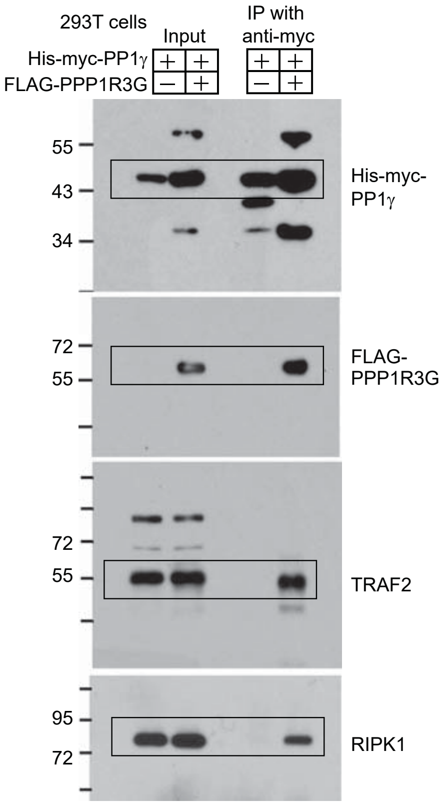

Fig 4c

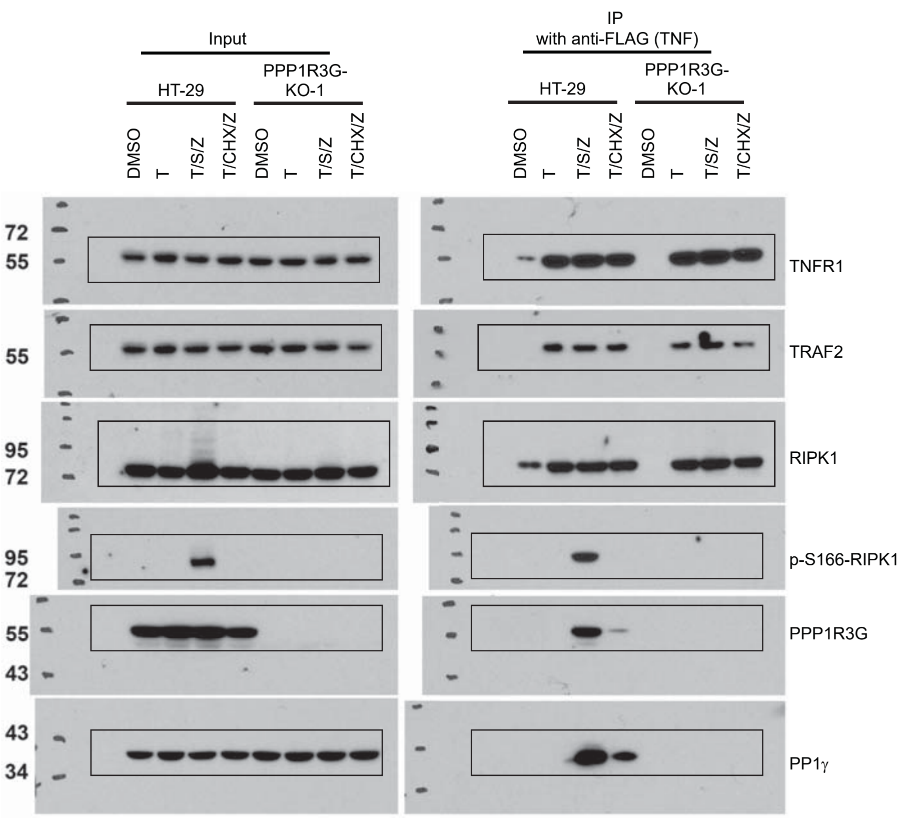

Fig 4b

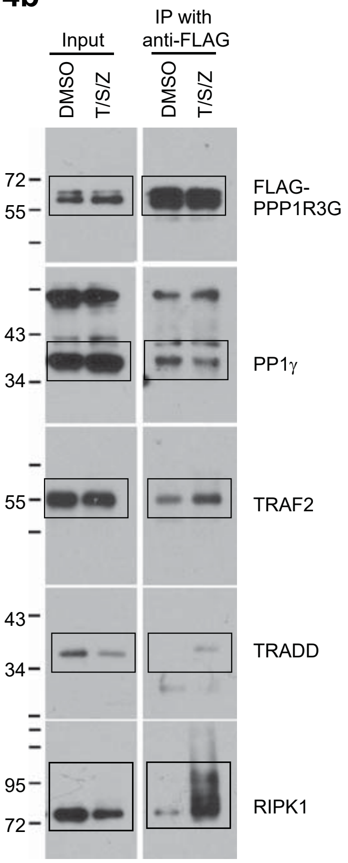

Fig 4d

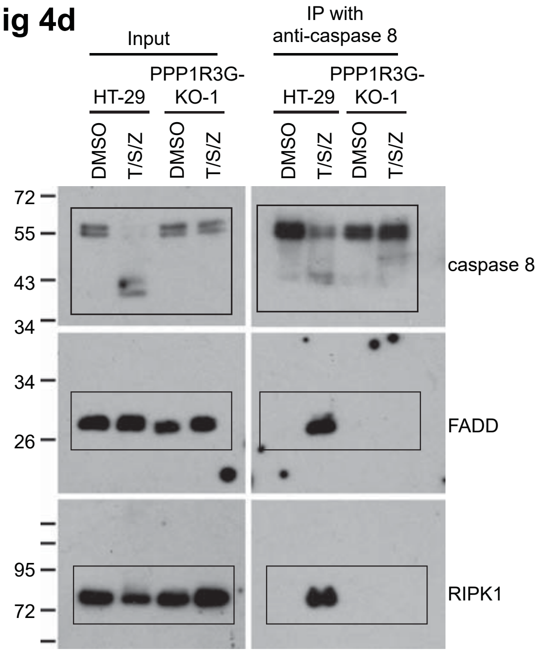

Supplementary figure 3. Source data immunoblots (continued).

Fig 5a

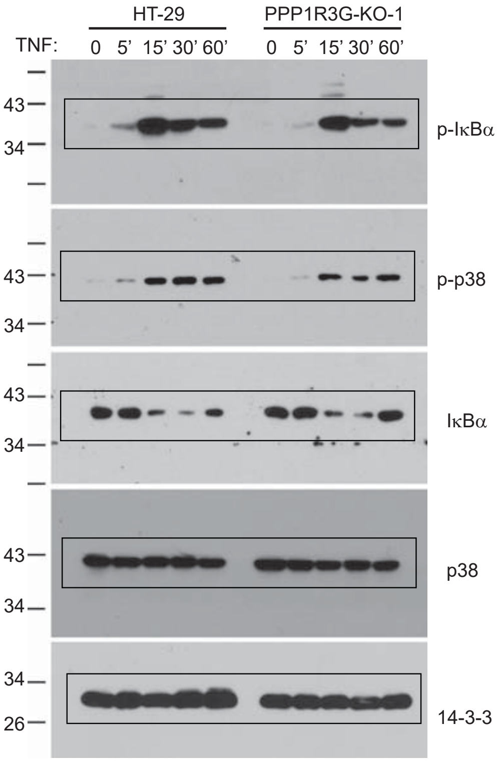

Fig 5c

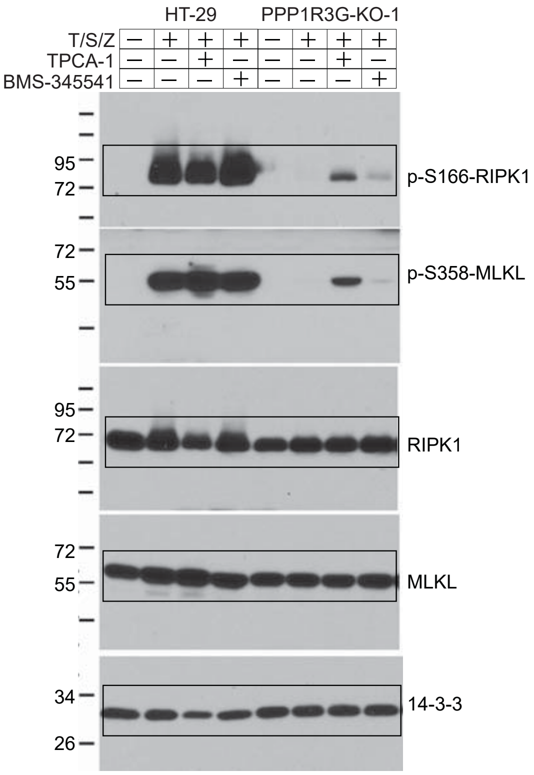

Fig 5d

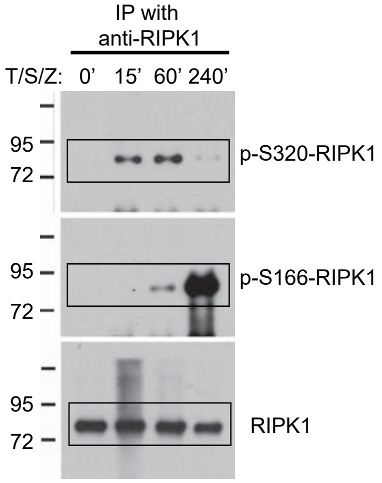

Fig 5e

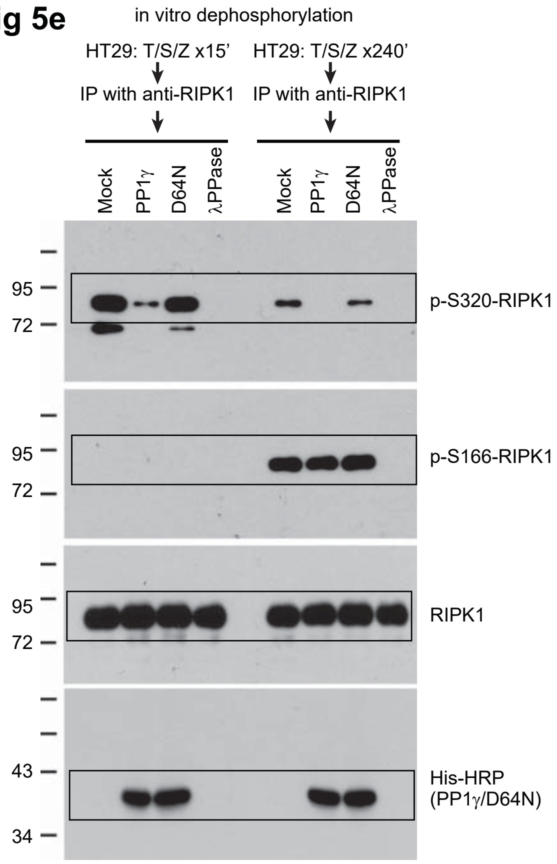

Supplementary figure 3. Source data immunoblots (continued).

Fig 6a

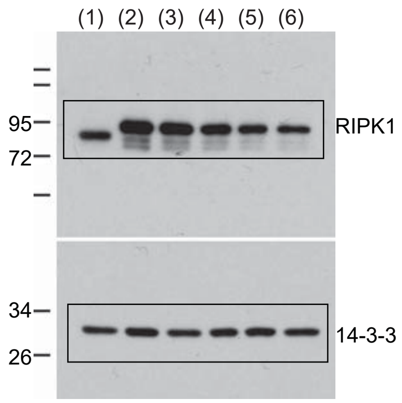

Fig 6b

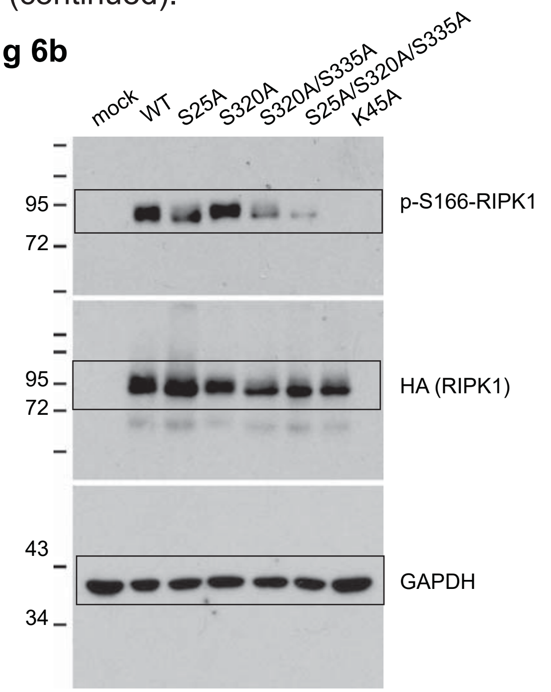

Fig 6c

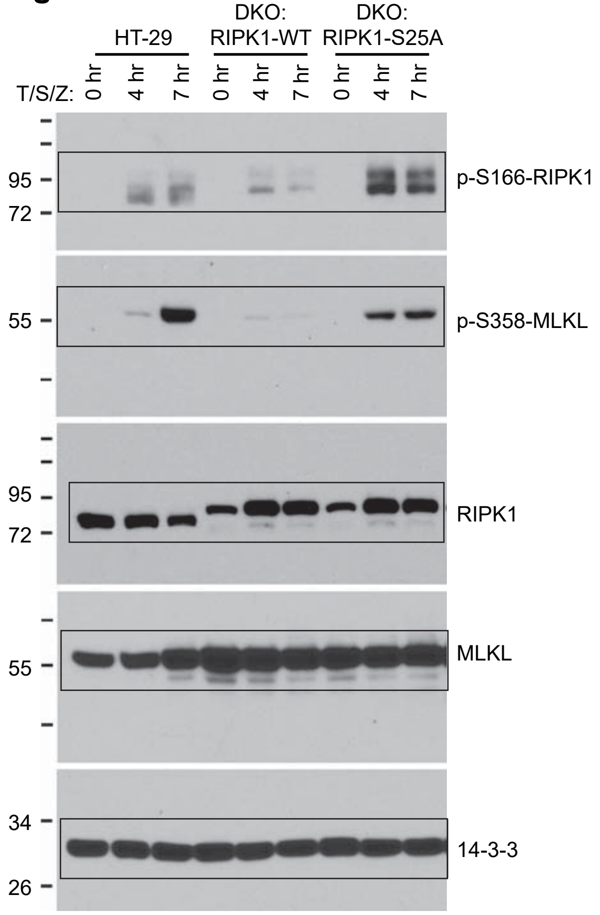

Fig 6e

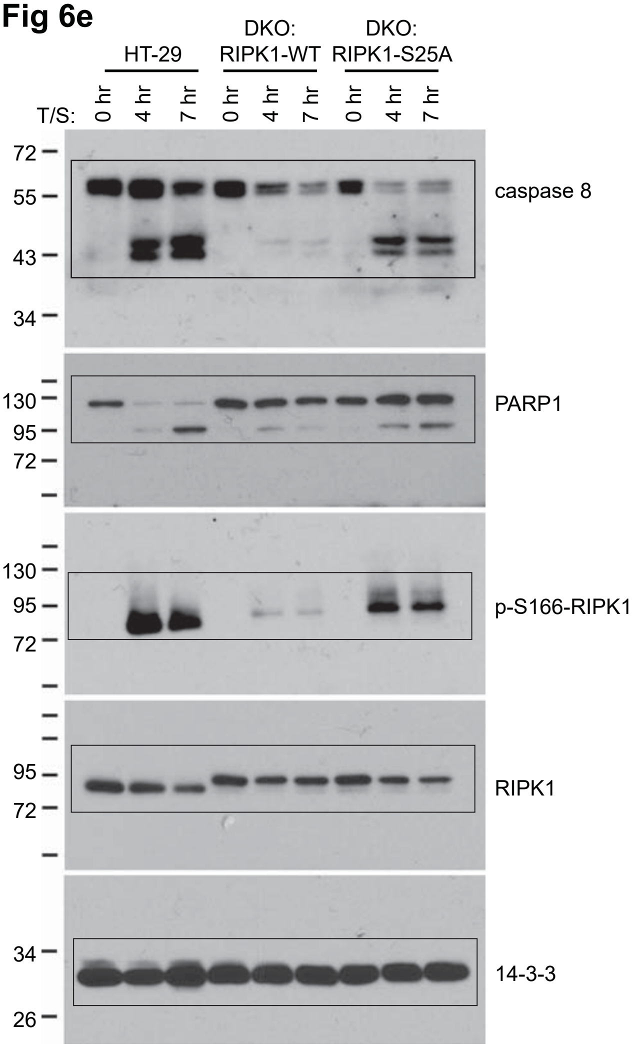

Supplementary figure 3. Source data immunoblots (continued).

Fig 7b

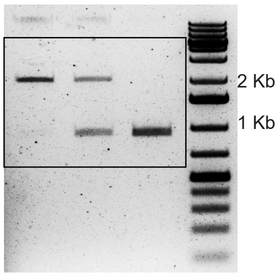

Fig 7c

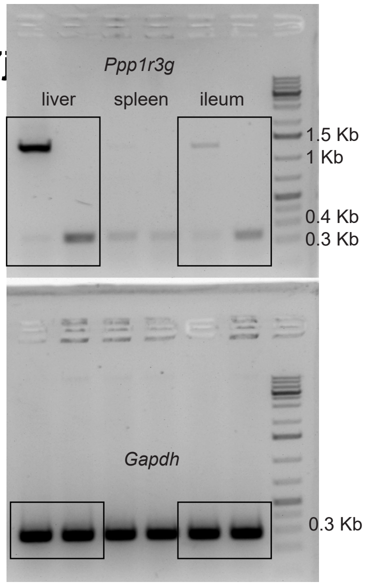

Fig 7e

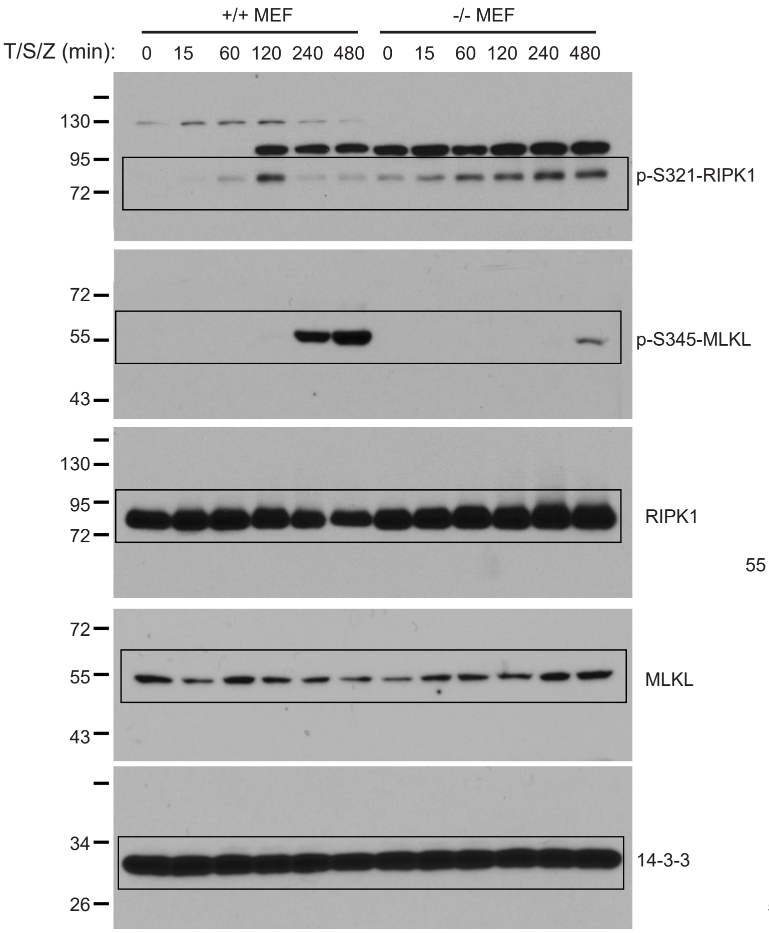

Fig 7j

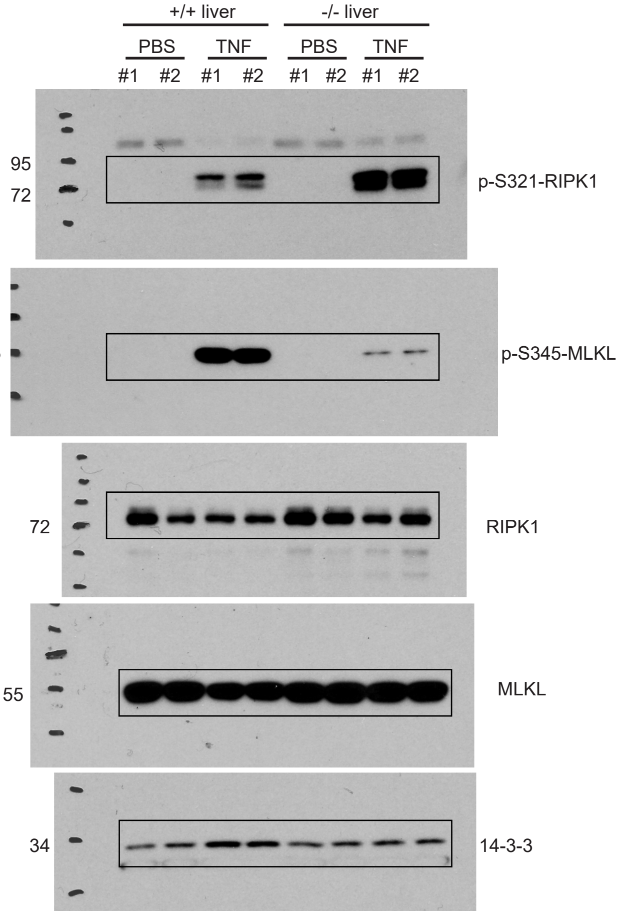

Supplement: Supplementary file 1 — Supplementary Information [file 41467_2021_27367_MOESM1_ESM.pdf]
