# Supplementary material for: RIPK1 dephosphorylation and kinase activation by PPP1R3G/PP1γ promote apoptosis and necroptosis
Source: Nat Commun. 2021 Dec 3;12:7067. doi: 10.1038/s41467-021-27367-5 (PMC8642546; doi:10.1038/s41467-021-27367-5)
Supplement: Supplementary file 3 — Description of Additional Supplementary Files [file 41467_2021_27367_MOESM3_ESM.docx]

**Description of Additional Supplementary Files**

File name: Supplementary Data 1

Description: Screening gene summary. Related to Figure 1. Positive genes are ranked according to the p-value. Number of positive sgRNA for each gene is listed in column D.

File name: Supplementary Data 2

Description: Normalized counts. Related to Figure 1. Normalized count for each sgRNA is listed for control and T/S/Z-treated samples. Median normalized counts of sgRNA were generated by MAGeCK^63^. 119,160 of total 119,462 (99.7%) sgRNA were detected in the control group, suggesting almost complete coverage of the library in the starting cell population.

File name: Supplementary Data 3

Description: Normalized count for non-targeting control guides. Related to Figure 1. Normalized count for non-targeting control guides is listed for control and T/S/Z-treated samples. Non-targeting guides are critical in evaluating the noise in the screen.

File name: Supplementary Data 4

Description: Library sgRNA for RIPK3 and MLKL. Related to Figure 1. All 6 sgRNA sequences for RIPK3 and MLKL are listed Their targeting sites in coding sequence (CDS) and transgene are provided. Four of five sgRNA targeting transgene RIPK3-DmrB and all five sgRNA targeting transgene MLKL-mCherry were highly enriched in T/S/Z-treated samples.

File name: Supplementary Data 5

Description: Sequences for secondary siRNA screen and cell viability results. Related to Supplementary Figure 2.

File name: Supplementary Data 6

Description: List of proteins precipitated with FLAG-PPP1R3G. Related to Figure 3a. Lysates from PPP1R3G-KO-1 and PPP1R3G-Rescue cells were subjected to anti-FLAG immunoprecipitation (IP) followed by mass spectrometry analysis. Abundance of each protein detected in KO and Rescue samples was listed and fold-enrichment was calculated accordingly.

File name: Supplementary Data 7

Description: Mass spec results for RIPK1 phospho-peptides. Related to Figure 5f. HT-29 cells were treated with TNF for 15 min and cell lysates were subjected to anti-RIPK1 IP. RIPK1-bound beads were then incubated with recombinant PP1γ and the final products were subjected to mass spectrometry analysis. The abundance of each RIPK1 peptide was listed. Total count was calculated by combing phospho-peptide count and non-phospho-peptide count for each peptide.
