# Supplementary material for: RIPK1 dephosphorylation and kinase activation by PPP1R3G/PP1γ promote apoptosis and necroptosis
Source: Nat Commun. 2021 Dec 3;12:7067. doi: 10.1038/s41467-021-27367-5 (PMC8642546; doi:10.1038/s41467-021-27367-5)
Supplement: Supplementary file 11 — Reporting Summary [file 41467_2021_27367_MOESM11_ESM.pdf]

Corresponding author(s): Zhigao Wang

Last updated by author(s): 10/25/2021

Reporting Summary

Nature Research wishes to improve the reproducibility of the work that we publish. This form provides structure for consistency and transparency in reporting. For further information on Nature Research policies, see our Editorial Policies and the Editorial Policy Checklist.

Statistics

For all statistical analyses, confirm that the following items are present in the figure legend, table legend, main text, or Methods section.

n/a	Confirmed	
☐	☒	The exact sample size (*n*) for each experimental group/condition, given as a discrete number and unit of measurement
☐	☒	A statement on whether measurements were taken from distinct samples or whether the same sample was measured repeatedly
☐	☒	The statistical test(s) used AND whether they are one- or two-sided<br>*Only common tests should be described solely by name; describe more complex techniques in the Methods section.*
☒	☐	A description of all covariates tested
☐	☒	A description of any assumptions or corrections, such as tests of normality and adjustment for multiple comparisons
☐	☒	A full description of the statistical parameters including central tendency (e.g. means) or other basic estimates (e.g. regression coefficient) AND variation (e.g. standard deviation) or associated estimates of uncertainty (e.g. confidence intervals)
☐	☒	For null hypothesis testing, the test statistic (e.g. *F*, *t*, *r*) with confidence intervals, effect sizes, degrees of freedom and *P* value noted<br>*Give P values as exact values whenever suitable.*
☒	☐	For Bayesian analysis, information on the choice of priors and Markov chain Monte Carlo settings
☒	☐	For hierarchical and complex designs, identification of the appropriate level for tests and full reporting of outcomes
☐	☒	Estimates of effect sizes (e.g. Cohen's *d*, Pearson's *r*), indicating how they were calculated

Our web collection on statistics for biologists contains articles on many of the points above.

Software and code

Policy information about availability of computer code

Data collection	No software was used for data collection.
Data analysis	Excel, GraphPad Prism 8.0

For manuscripts utilizing custom algorithms or software that are central to the research but not yet described in published literature, software must be made available to editors and reviewers. We strongly encourage code deposition in a community repository (e.g. GitHub). See the Nature Research guidelines for submitting code & software for further information.

Data

Policy information about availability of data

All manuscripts must include a data availability statement. This statement should provide the following information, where applicable:

- Accession codes, unique identifiers, or web links for publicly available datasets
- A list of figures that have associated raw data
- A description of any restrictions on data availability

CRISPR screening data generated in this study have been deposited to GEO with an identification number GSE176422 (https://www.ncbi.nlm.nih.gov/geo/query/acc.cgi?acc=GSE176422). All other data generated during the current study are included in this published article and its supplementary information files.

Field-specific reporting

Please select the one below that is the best fit for your research. If you are not sure, read the appropriate sections before making your selection.

☒ Life sciences ☐ Behavioural & social sciences ☐ Ecological, evolutionary & environmental sciences

For a reference copy of the document with all sections, see nature.com/documents/nr-reporting-summary-flat.pdf

Life sciences study design

All studies must disclose on these points even when the disclosure is negative.

Sample size	For the cell survival assay, Data are represented as mean ± SD of n=3 biologically independent samples. At least 3 independent experiments were performed. For mice SIRS experiments, GraphPad Prism 8 was used to determine the sample size and statistical significance.
Data exclusions	There were no data excluded for statistical analysis.
Replication	All data were independently repeated with similar results.
Randomization	Samples were not randomized. Positive and negative controls were included for all experiments.
Blinding	Mouse survival and temperature measurement as well as blood samples were done blindly.

Reporting for specific materials, systems and methods

We require information from authors about some types of materials, experimental systems and methods used in many studies. Here, indicate whether each material, system or method listed is relevant to your study. If you are not sure if a list item applies to your research, read the appropriate section before selecting a response.

Materials & experimental systems

n/a	Involved in the study	
☐	☒	Antibodies
☐	☒	Eukaryotic cell lines
☒	☐	Palaeontology and archaeology
☐	☒	Animals and other organisms
☒	☐	Human research participants
☒	☐	Clinical data
☒	☐	Dual use research of concern

Methods

n/a	Involved in the study	
☒	☐	ChIP-seq
☒	☐	Flow cytometry
☒	☐	MRI-based neuroimaging

Antibodies

Antibodies used	Anti-FLAG M2 (F-1804) and anti-myc (A7470) antibody and affinity gel (Sigma), anti-RIPK1 (BD551042), anti-phospho-S166 of RIPK1 (Cell Signaling, 65746S), anti-phsopho-S320 of RIPK1 (gift from Dr. Pascal Meier), anti-phospho-S321 of mouse RIPK1 (Cell Signaling, 83613), anti-human MLKL (GeneTex, GTX107538), anti-mouse MLKL (Aviva Systems Biology, OAAB10571), anti-phospho-S358 of MLKL (Abcam, ab187091), anti-phospho-S345 of mouse MLKL (Cell Signaling, 37333S), anti-TNFR1 (Santa Cruz, SC-8436), anti-TRADD (BD, 610572), anti-TRAF2 (Cell Signaling, 4724S), anti-cIAP-2 (BD, 51-90000062), anti-PP1gamma (Prosci, 58-851), anti-caspase 8 (Santa Cruz, SC-6136 and SC-81656), anti-FADD (Enzo, AAM212-E), anti-PARP-1 (BD, 65196E), anti-LDH (Abcam, ab53292) and anti-14-3-3 (Santa Cruz, SC-629). Rabbit anti-PPP1R3G was generated by immunizing with recombinant full-length protein produced in E. Coli (Pacific Immunology).
Validation	Commercial antibodies were validated by the manufactures and some were validated with lysates from individually gene-silenced cells. The anti-PPP1R3G antibody was validated by Western blotting using lysates from PPP1R3G-knockout cells.

Eukaryotic cell lines

Policy information about cell lines

Cell line source(s)	HAP1 cells (Horizon Genomics) were cultured in IMDM supplemented with 10% FBS. HT-29 (ATCC) and MEF cells were cultured in DMEM (high glucose) supplemented with 10% FBS. (1) HAP1:RIPK3:MLKL cells. HAP1 cells were stably transfected with CMV-driven Tet Repressor (TetR, selected with 10 µg/ml Blasticidin), followed by stable expression of Dox-inducible RIPK3-DmrB fusion protein (selected with 0.5 mg/ml G418) and Dox-inducible MLKL-mCherry fusion protein (selected with 1 µg/ml puromycin). The transgene expression was induced with 50 ng/mL Dox for 24 hours. (2) Knockout cell lines. CRISPR-mediated KO cell lines were generated as described before 60. Briefly, gene-specific targeting oligos were cloned into the LentiCRISPR V2 vector (Addgene 52961), which was co-transfected with pMD2.G (Addgene 12259) and psPAX2 (Addgene

	12260) into 293T cells to produce lentiviruses. Parental cells were then transduced with the viruses and single clones were selected with 1 μg/ml puromycin. RIPK1 was knocked out in PPP1R3G-KO cells to establish the PPP1R3G/RIPK1-DKO cell line. Gene knockout was confirmed by Western blotting and sequencing. The following targeting sequences were used. PPP1R3G, TGCGCTACACCTTTACCGAG; and RIPK1, TGGAAAAGGCGTGATACACA. (3) Rescue cell lines. Tagged constructs with sense mutations at the sgRNA-targeting sites were stably expressed in the KO cells by lentiviral transduction to generate the rescue cell lines. (4) MEF cells. E12.5 to E14.5 embryos were harvested and digested with trypsin. Fibroblast cells were plated and passed every three days. The experiments were done within 5 passages.
Authentication	HAP1 (Horizon Genomics), HT-29 (ATCC) and 293T (ATCC) cells were authenticated by the commercial sources. All other genetically modified cell lines were confirmed by Western blotting.
Mycoplasma contamination	No mycoplasma contamination was detected.
Commonly misidentified lines (See ICLAC register)	No commonly misidentified lines were used.

Animals and other organisms

Policy information about studies involving animals; ARRIVE guidelines recommended for reporting animal research

Laboratory animals	PPP1R3G-KO mice were backcrossed to C57BL/6 background. For SIRS model, 8-14 weeks old, age and sex-matched mice were injected through tail vein with mouse TNF (0.3 μg TNF/g body weight).
Wild animals	No wild animals were used for this study.
Field-collected samples	No field-collected samples were used.
Ethics oversight	Animal work described in this manuscript has been approved and conducted under the oversight of the Institutional Animal Care and Use Committee at UT Southwestern.

Note that full information on the approval of the study protocol must also be provided in the manuscript.
